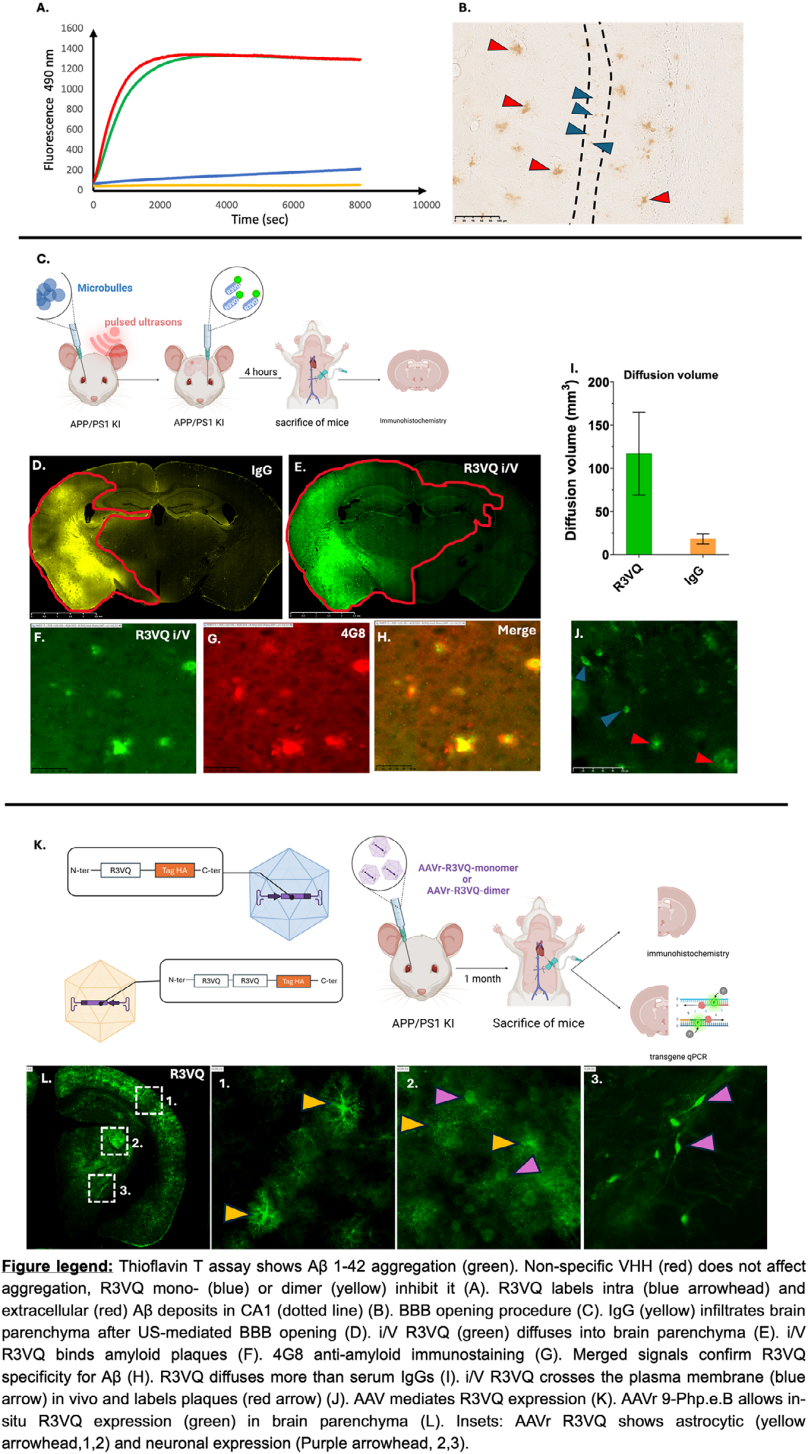# Enhanced anti‐Aß immunotherapies using nanobodies in an AD mouse model

**DOI:** 10.1002/alz70859_103908

**Published:** 2025-12-25

**Authors:** Alexandre Florent Trotier, Jean David Randrianaly, Amandine Géraudie, Sylvie Jacquot, Françoise Piguet, Sylvie Bay, Pierre Lafaye, Benoit Delatour, Alzheimer's and Prion diseases

**Affiliations:** ^1^ Paris Brain Institute (ICM), Paris France; ^2^ Paris Brain Institute (ICM), Inserm, CNRS, Sorbonne University, Paris, Ile de France France; ^3^ Pasteur Institute, Paris France

## Abstract

**Background:**

Alzheimer’s disease (AD), the leading cause of dementia, is characterized by amyloid‐beta (Aβ) peptide accumulation, which forms plaques that trigger inflammation, synaptic loss, and neuronal death. Although monoclonal antibodies (mAbs) targeting Aβ aggregates have shown success in reducing plaques, their effect on cognitive decline is limited, potentially due to the challenge posed by the blood‐brain barrier (BBB) and brain structure. Camelid nanobodies (VHHs), due to their small size (15 kDa) and single‐domain structure, can more effectively penetrate the BBB, diffuse within the brain, and bind difficult‐to‐reach epitopes, such as those on Aβ plaques or within the intracellular compartment. To enhance the delivery of anti‐Aβ VHHs, two strategies were developed: transient BBB opening and brain in situ production via viral vectors. This study aims to develop and assess two novel immunotherapy approaches using the anti‐Aβ VHH R3VQ in an AD mouse model.

**Method:**

R3VQ was optimized into monomeric, dimeric, and Fc‐conjugated formats. In vivo efficacy was evaluated using APP‐PS1‐KI mice, which develop both intracellular and extracellular Aβ aggregates. The diffusion of R3VQ was assessed through stereotaxic injections of R3VQ variants into the dorsal hippocampus, followed by histological analysis of brain tissue. To transiently open the BBB, microbubbles and low‐intensity pulsed ultrasound (LIPU) were used in combination with intravenous (i/V) R3VQ injections, and brain bioavailability was analyzed four hours post‐injection. Additionally, AAVr vectors were used to express monomeric or dimeric R3VQ in the brain, and expression was assessed using qPCR and immunofluorescent staining.

**Result:**

In vitro assays confirmed that R3VQ effectively recognizes fibrillar and soluble Aβ, inhibiting fibril formation. In vivo, both monomeric and dimeric R3VQ successfully labeled Aβ deposits and diffused widely in the brain compared to conventional IgGs. Preliminary results from the enhanced immunotherapy strategies show that R3VQ can cross the BBB, bind amyloid plaques, and enter neuronal cells following LIPU‐induced BBB opening. AAV delivery of R3VQ resulted in robust astrocytic expression in the brain. Ongoing analyses aim to evaluate the therapeutic potential of these approaches.

**Conclusion:**

This study explores innovative passive immunotherapy strategies targeting Aβ, with potential to advance AD treatment and deepen understanding of the disease’s pathophysiology.